# Supplementary material for: Pilot study of the relation between various dynamics of avatar experience and perceptual characteristics
Source: PeerJ Comput Sci. 2024 May 21;10:e2042. doi: 10.7717/peerj-cs.2042 (PMC11157551; doi:10.7717/peerj-cs.2042)
Supplement: Supplemental Information 2 [file peerj-cs-10-2042-s002.pdf]

Appendix

In this study, we explored the impact of altering SoA, SoO, and CL through delays, including time lags and phase delays, as well as by considering the involved overshoot characteristics and movement intensity. Additionally, we examined their discrimination threshold for various settings using the specified avatar.

In a preliminary experiment, the study investigated how a change in SoO, SoA, and CL by manipulating avatar settings and the discrimination threshold of these setting parameters were related.

METHODS

Details of the used methods are present in the main manuscript. Method details considered as appendix material are as follows.

There were "comparison settings": gain [−] ($K_i = K_{\mathrm{NM}} \cdot K_{\mathrm{MS}}$) is $\{K_1,\ K_2,\ K_3,\ K_4\}$=$\{150,\ 300,\ 400,\ 600\}$, damping coefficient [−] ($\zeta_i = \zeta_{\mathrm{MN}} = \zeta_{\mathrm{MS}}$) is $\{\zeta_1, \zeta_2, \zeta_3, \zeta_4\} = \{0.2,\ 0.45,\ 0.7,\ 0.95\}$. Meanwhile, the fourth questionnaire was designed to investigate the discrimination thresholds in which participants were asked whether there was a difference in the sensations of avatar control experience (whether they feel avatar movement as a "lack of movement" (−) or "hyper responsive movement" (+)), and rated their experience on a worst to best scale from "−3 to +3" compared to the standard setting (0) in "0.25" increments.

Examples of System Response

Examples of the avatar system's responses to the NMSS model inputs are shown in Figure 10. As indicated in the figure, gain contributes to movement intensity, damping coefficient contributes to overshoot respectively.

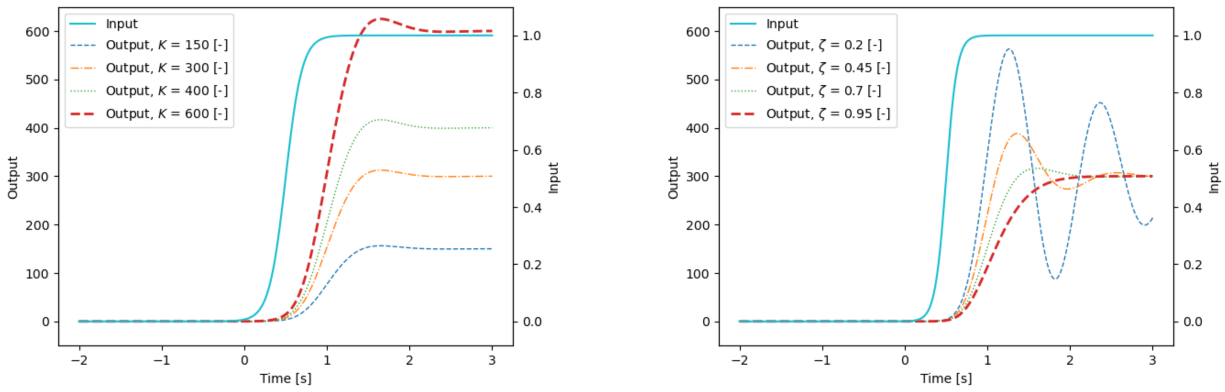

Figure 10: Pattern of each setting: left: gain, right: damping coefficient

RESULT

The values shown in each graph are the averaged value for all participants and its standard deviation. The values shown in the respective tables are the effect size of the multiple comparisons of the respective averaged values.

Discrimination threshold

The respective evaluated values and effect size with an asterisk indicate the size of those shown in Figure 11 and Table 4.

- At whole parameters, evaluated values were close to zero in dummy setting (K_2, ω_{np2}, ζ_3, L_1).
- For gain, the larger the comparison setting value, the more positive (felt "hyper responsive movement") the evaluated value was. In addition, all of the effect sizes were "large."
- For natural angular frequency, the smaller the comparison setting value, the more negative (felt "lack of movement") the evaluated value was. The effect size for ω_{np1} - ω_{np2} was "medium," and all other effect sizes were "large."
- For the damping coefficient, the larger the comparison setting value, the more negative the evaluated value (felt "lack of movement"). In addition, all of the effect sizes were "large."
- In dead time, when the comparison setting value shifted from L_1 to L_2, the evaluated value was biased toward positive (felt "hyper responsive movement") and thereafter toward negative (felt "lack of movement"). The effect size for L_1 - L_2 and L_3 - L_4 were "medium," while the effect size for L_2 - L_3 and L_2 - L_4 were "large."

Table 4: Discrimination threshold: effect sizes

	K_2	K_3	K_4
K_1	.82**	.90**	.91**
K_2		.66**	.72**
K_3			.77**

	ω_{np2}	ω_{np3}	ω_{np4}
ω_{np1}	.48*	.93**	.96**
ω_{np2}		.75**	.88**
ω_{np3}			.87**

	ζ_2	ζ_3	ζ_4
ζ_1	.87**	.93**	.98**
ζ_2		.68**	.92**
ζ_3			.89**

	L_2	L_3	L_4
L_1	.46*	.04	.28
L_2		.87**	.53**
L_3			.31*

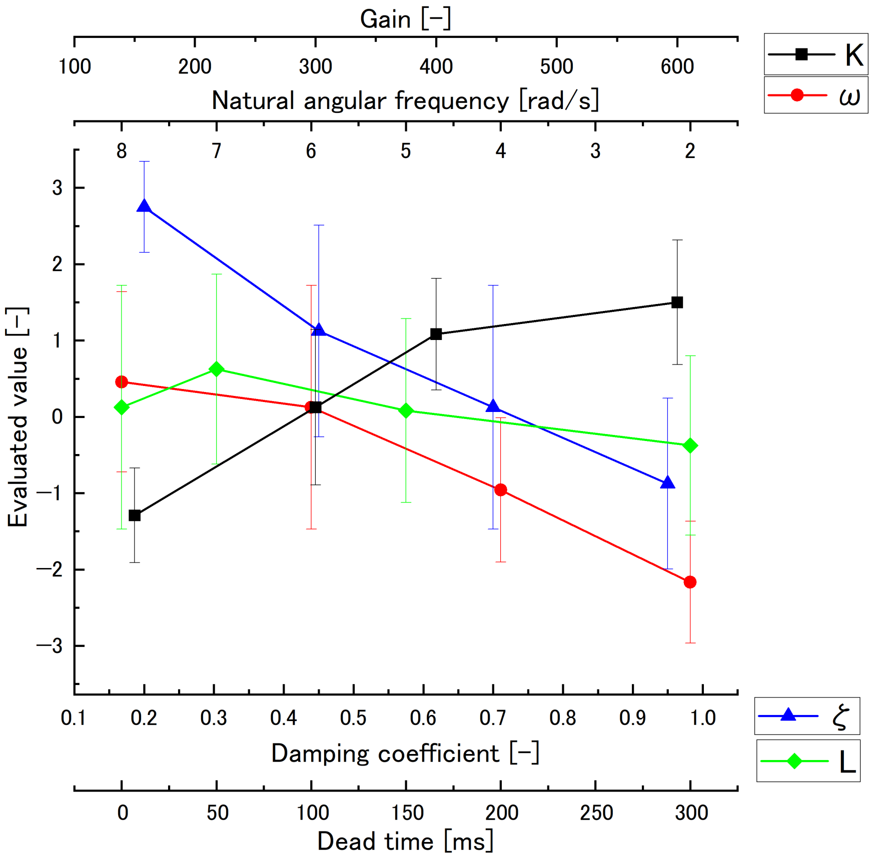

Figure 11: Discrimination threshold: evaluated value; natural angular frequency axis was reversed to be consistent with the dead time direction. The smaller the natural angular frequency, the larger the delay. However, because natural angular frequency range was narrow, its axis is not log scale.

Gain

The evaluated value and effect size in gain are shown in Figure 12 and Table 5.

- SoO values tended to be convex downward. The effect size of $K_3 - K_4$ was "medium."
- SoA values did not change effectively, regardless of the change in comparison setting value. Meanwhile, there was no effect size indicating "medium" or "large."
- CL values did not change effectively in the range of $K_2 \leq K_i \leq K_4$, but fell at K_1. The effect sizes of $K_1 - K_2$ and $K_1 - K_3$ were "medium."

Table 5: Gain in the preliminary experiment: effect sizes (upper: SoO, middle: SoA, and lower: CL)

	K_2	K_3	K_4
K_1	.26	.22	.00
K_2		.10	.25
K_3			.32*

	K_2	K_3	K_4
K_1	.11	.03	.14
K_2		.15	.02
K_3			.17

	K_2	K_3	K_4
K_1	.41*	.36*	.26
K_2		.06	.06
K_3			.03

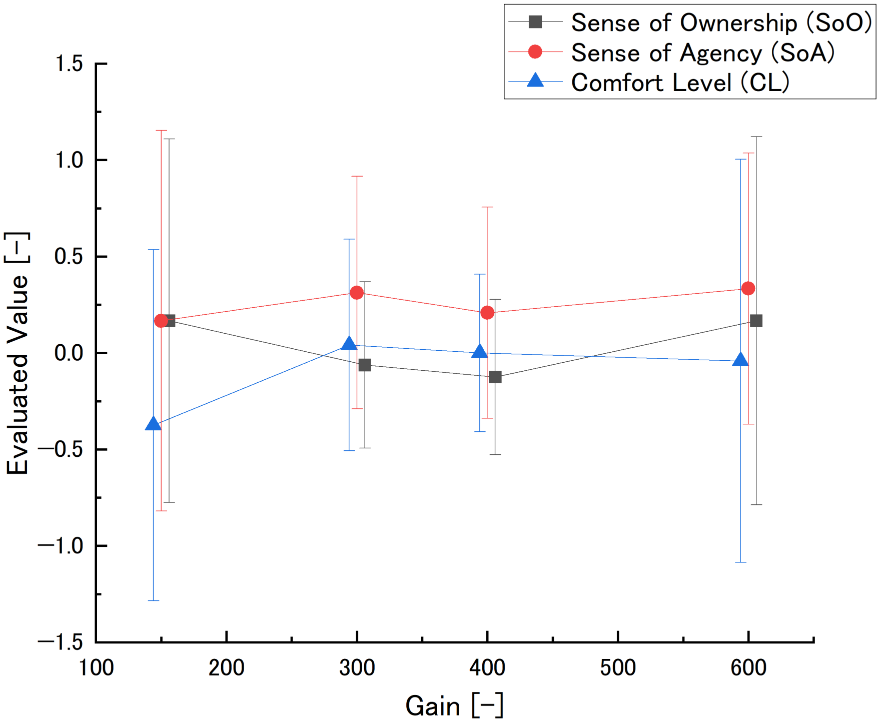

Figure 12: Gain in the preliminary experiment: evaluated value

Damping Coefficient

The evaluated values and effect sizes for the damping coefficient are shown in Figure 13 and Table 6.

- SoO values increased as the comparison setting value increased. The effect size of $\zeta_3 - \zeta_4$ was "medium," and all other effect sizes were "large."
- SoA values increased as the comparison setting value increased. All effect sizes, except for $\zeta_3 - \zeta_4$ were "large."

- CL values increased with increasing value in the range $\zeta_1 \leq \zeta_i \leq \zeta_3$ and decreased with increasing value in the range $\zeta_3 \leq \zeta_i \leq \zeta_4$. The effect size of $\zeta_2 - \zeta_4$ was "medium" and that of $\zeta_1 - \zeta_2$, $\zeta_1 - \zeta_3$, $\zeta_1 - \zeta_4$ and $\zeta_2 - \zeta_3$ were "large."

Table 6: Damping coefficient in the preliminary experiment: effect sizes (upper: SoO, middle: SoA, and lower: CL)

	ζ_2	ζ_3	ζ_4
ζ_1	.62**	.77**	.79**
ζ_2		.80**	.77**
ζ_3			.46*

	ζ_2	ζ_3	ζ_4
ζ_1	.85**	.87**	.88**
ζ_2		.64**	.56**
ζ_3			.12

	ζ_2	ζ_3	ζ_4
ζ_1	.86**	.94**	.93**
ζ_2		.83**	.54*
ζ_3			.11

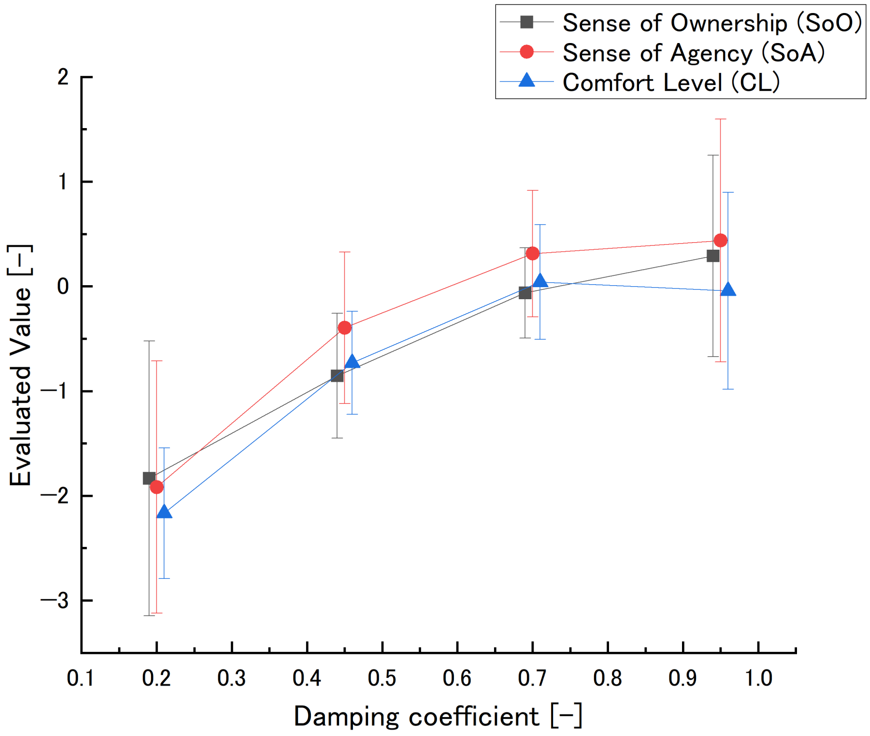

Figure 13: Damping coefficient in the preliminary experiment: evaluated value

DISCUSSION

Discrimination threshold

In gain, all effect sizes for K_3, which is the same as those in the standard setting, are “large,” suggesting the participants were able to discriminate each setting compared to the standard setting situation. This suggests that at least 100, the minimum change in this experiment, can be discriminated in gain.

In the natural angular frequency, all effect sizes for ω_{np2}, which is the same as those in the standard setting, were either “medium” or “large,” suggesting the participants were able to discriminate between each comparison setting compared to the standard setting situation. This suggests the participants were able to discriminate at least 2, which is the natural angular frequency minimum change in this experiment. The fact that the effect size of $\omega_{\mathrm{np1}} - \omega_{\mathrm{np2}}$ was smaller than that of $\omega_{\mathrm{np2}} - \omega_{\mathrm{np3}}$ and $\omega_{\mathrm{np2}} - \omega_{\mathrm{np4}}$ suggests that it is easier to discriminate smaller changes than larger changes than one is able to in the standard setting situation.

In the damping coefficient, all effect sizes for ζ_3, which is the same as those in the standard setting, are “large,” suggesting the participants were able to discriminate each setting compared to the standard setting situation. This suggests that at least 0.25, the minimum change in this experiment, can be discriminated for the damping coefficient.

In dead time, only the $L_1 - L_2$ effect size for L_1, which is the same as that in the standard setting, shows a “medium” effect size, and none of the effect sizes show a “large” effect size. $L_1 - L_3$ and $L_1 - L_4$, which are larger changes than $L_1 - L_2$, are smaller than $L_1 - L_2$. This result has two points. First, this experiment made only “positive delay” but participants answered “hyper responsive movement” at L_2 with “medium” effect. Second, previous study indicated that above about 200 ms delay from the real body can be sufficiently detected by the participant [1]. However, in this study, L_4 was not detected. Related to the first point, there is a phenomenon called “intentional binding,” in which the sensation of perception is shortened when the participant feels a SoA [2]. In Figure 7, L_2 has maximum SoA and CL. It is considered that these caused “intentional binding” and participants answered “hyper responsive movement” at L_2. For the second point, there were some different points from the previous study, e.g., target body part (it was finger in previous study) and how to ask question (synchronicity between real finger and finger video image was asked in previous study). These variations could have possibly made a difference in the results.

Gain

In the SoO values, the effect sizes of $K_1 - K_2$, $K_2 - K_3$, and $K_3 - K_4$, which compared adjacent settings, only the effect size of $K_3 - K_4$ shows a “medium” value, while the other two are “small,” and $K_2 - K_4$ shows a “small” in spite of K_2 being the same as the standard setting. It indicates that K_3 and K_4 are varied around the standard setting. Therefore, it is considered that SoO has no trends in this range of gain.

SoA values did not change effectively regardless of the change in the comparison settings. However, when looking at the effect sizes of $K_1 - K_2$, $K_2 - K_3$, and $K_3 - K_4$, which compare adjacent comparison settings, there were no effect sizes that indicated “medium” or “large,” while no other combination has “medium” or “large” effect. Therefore, there is no trend at SoA.

CL values increased from K_1 to K_2, but did not change effectively in the range of $K_2 \leq K_i \leq K_3$. Looking at

the effect sizes for $K_1 - K_2$, $K_2 - K_3$, and $K_3 - K_4$, each of which compares adjacent comparison settings, only $K_1 - K_2$ showed anything greater than "medium." Therefore, the tendency for the value to increase from K_1 to K_2 may be considered effective.

The range of gain in this study suggested that CL was impaired when gain was small, but gain had no effect on SoO or SoA. Gain is a parameter of movement intensity. Above a certain intensity, SoO, SoA, and CL are not affected, while only comfort is impaired at the lower intensity than a certain intensity. This suggests that CL may be a more acute questionnaire to the perceptions of changes in the target behavior than SoA.

Damping Coefficient

SoO increased as the comparison setting value increased. The effect size of $\zeta_1 - \zeta_2$, $\zeta_2 - \zeta_3$, and $\zeta_3 - \zeta_4$, which compare adjacent comparison settings, respectively shows "large," "large," and "medium."

SoA increased as the comparison setting value increased. The effect sizes of $\zeta_1 - \zeta_2$, $\zeta_2 - \zeta_3$, and $\zeta_3 - \zeta_4$, which compare adjacent comparison settings, respectively show "large," "large," and "small." Thus, this trend in the range of $\zeta_1 \leq \zeta_i \leq \zeta_3$ may be effective. However, this trend in the range of $\zeta_3 \leq \zeta_i \leq \zeta_4$ is hardly effective. In other words, SoA increased by increasing the damping coefficient in the low damping coefficient area, but has no effect on SoA in the high damping coefficient area.

CL increased with increasing value in the range of $\zeta_1 \leq \zeta_i \leq \zeta_3$ and decreased with increasing value in the range of $\zeta_3 \leq \zeta_i \leq \zeta_4$. The effect sizes of $\zeta_1 - \zeta_2$, $\zeta_2 - \zeta_3$, and $\zeta_3 - \zeta_4$, which compare adjacent comparison settings, respectively, show "large," "large," and "small." Thus, this trend in the of range $\zeta_1 \leq \zeta_i \leq \zeta_3$ may be effective. However, this trend in the range of $\zeta_3 \leq \zeta_i \leq \zeta_4$ is hardly effective. In other words, CL increased by increasing the damping coefficient in the low damping coefficient area, but has no effect on CL in the high damping coefficient area. The results of SoO and CL indicate that these have the possibility that the trend of generating SoO in the damping coefficient differs from SoA and CL.

The damping coefficient is a parameter of overshoot. These results indicate that the low damping coefficient, which cause a larger overshoot, make SoO, SoA, and CL decrease, while the high damping coefficient, which causes a smaller overshoot, make SoO, SoA and CL increase. However, it indicates that overshoot affects only SoO in the low overshoot area. These results indicate that it has the possibility that the trend of generating SoO in overshoot differs from SoA and CL.

SoA at Dummy Setting

Similar to natural angular frequency and dead time, for gain and damping coefficient, SoA was higher than 0 in the dummy setting, which is the same case as in the standard setting. This suggests that SoA may be improved by repetition compared to the other perceptual characteristics, since the participants always experience the avatar in each setting after experiencing the standard setting due to the experimental protocol. In contrast, CL is introduced as the alternative parameter of limb heaviness in order to allow questions not only about the feeling of heaviness, but also about the feeling of lightness, based also on reports that comfort is related to SoA [3]. SoA is changed from this result and CL may be a more acute questionnaire method in some cases.

References

[1] M. Osumi, S. Nobusako, T. Zama, N. Yokotani, S. Shimada, T. Maeda, and S. Morioka. “The relationship and difference between delay detection ability and judgment of sense of agency.” In: *PLOS ONE.* 14.7 (2019).

[2] S. Kasahara, J. Nishida, and P. Lopes. “Preemptive action: accelerating human reaction using electrical muscle stimulation without compromising agency.” In: *Proceedings of the 2019 CHI Conference on Human Factors in Computing Systems.* 2019, pp. 1–15.

[3] M. Mishima, K. Hayashida, Y. Fukasaku, R. Ogata, K. Ohsawa, K. Iwai, W. Wen, and S. Morioka. “Adaptability of the sense of agency in healthy young adults in sensorimotor tasks for a short term.” In: *Behavioral Sciences.* 13.2 (2023), p. 132.
